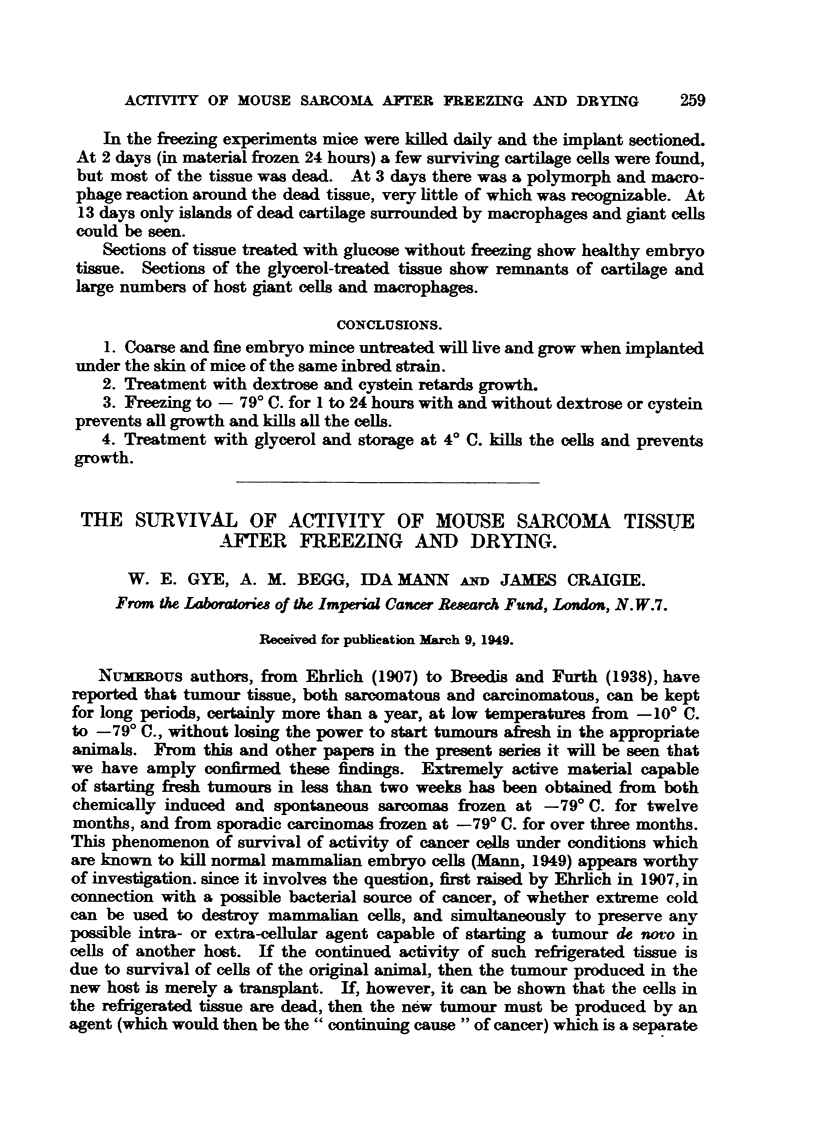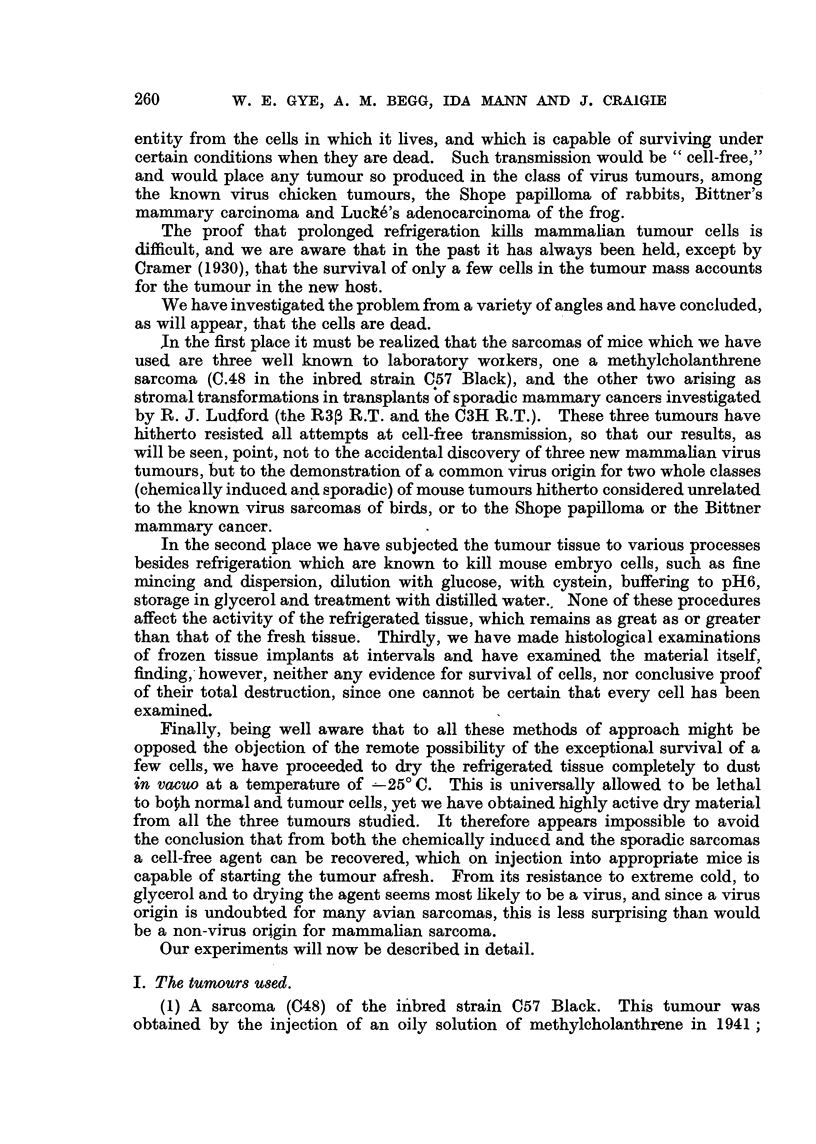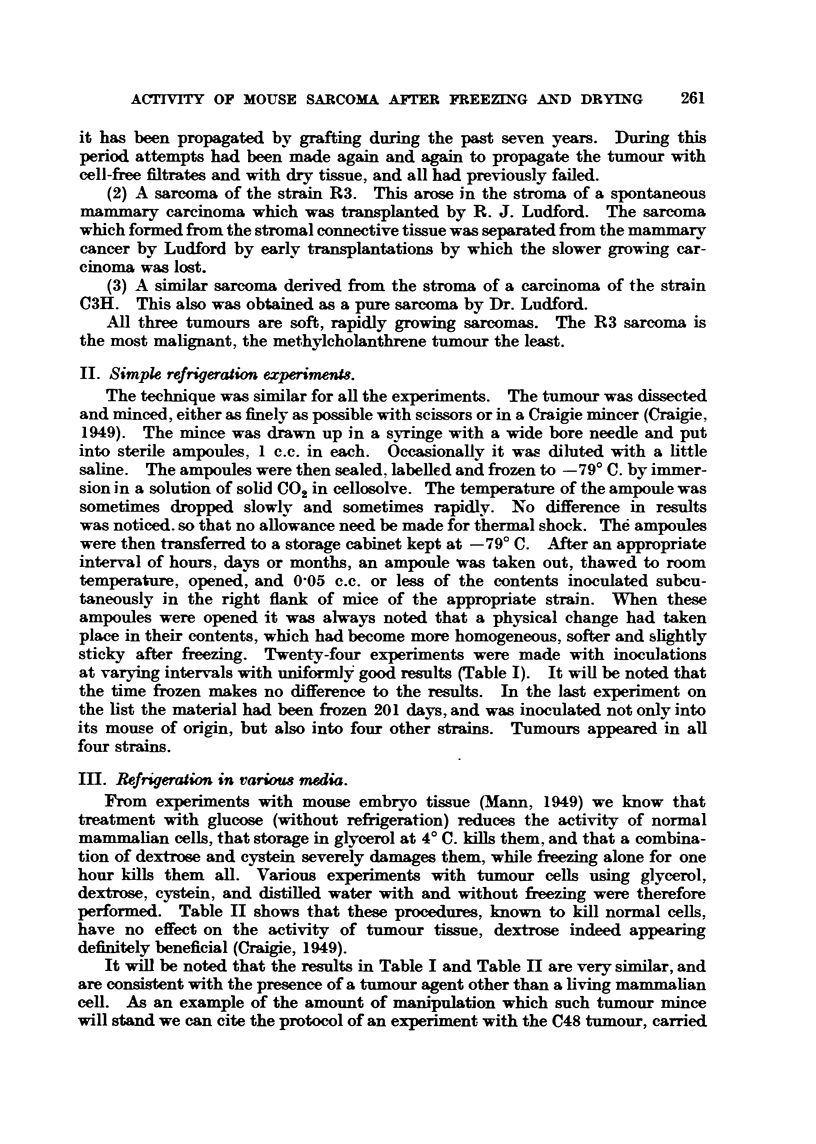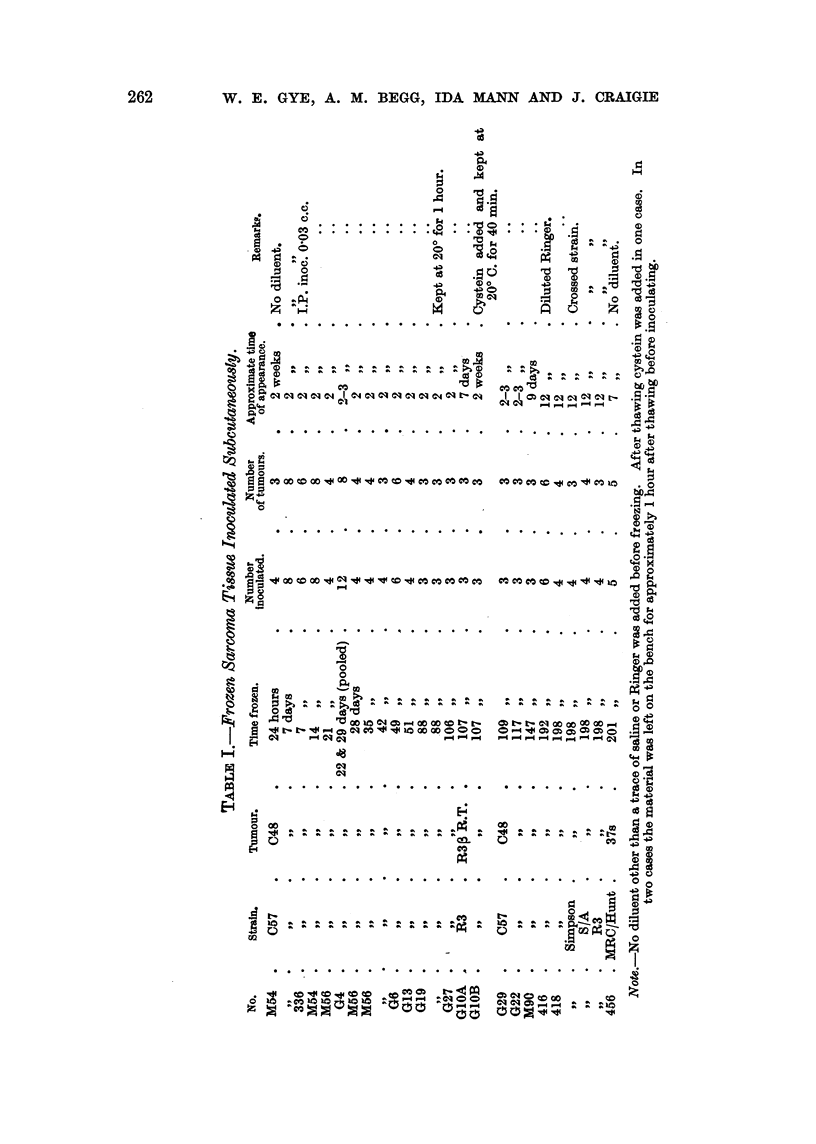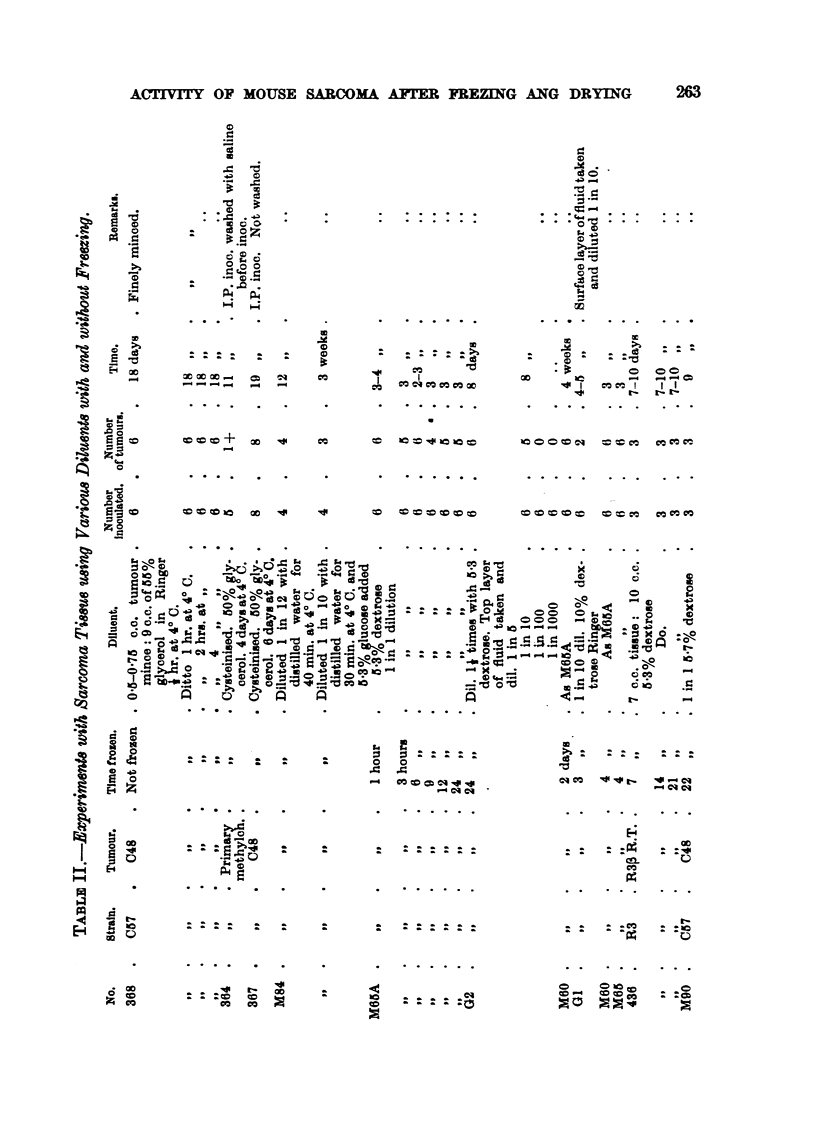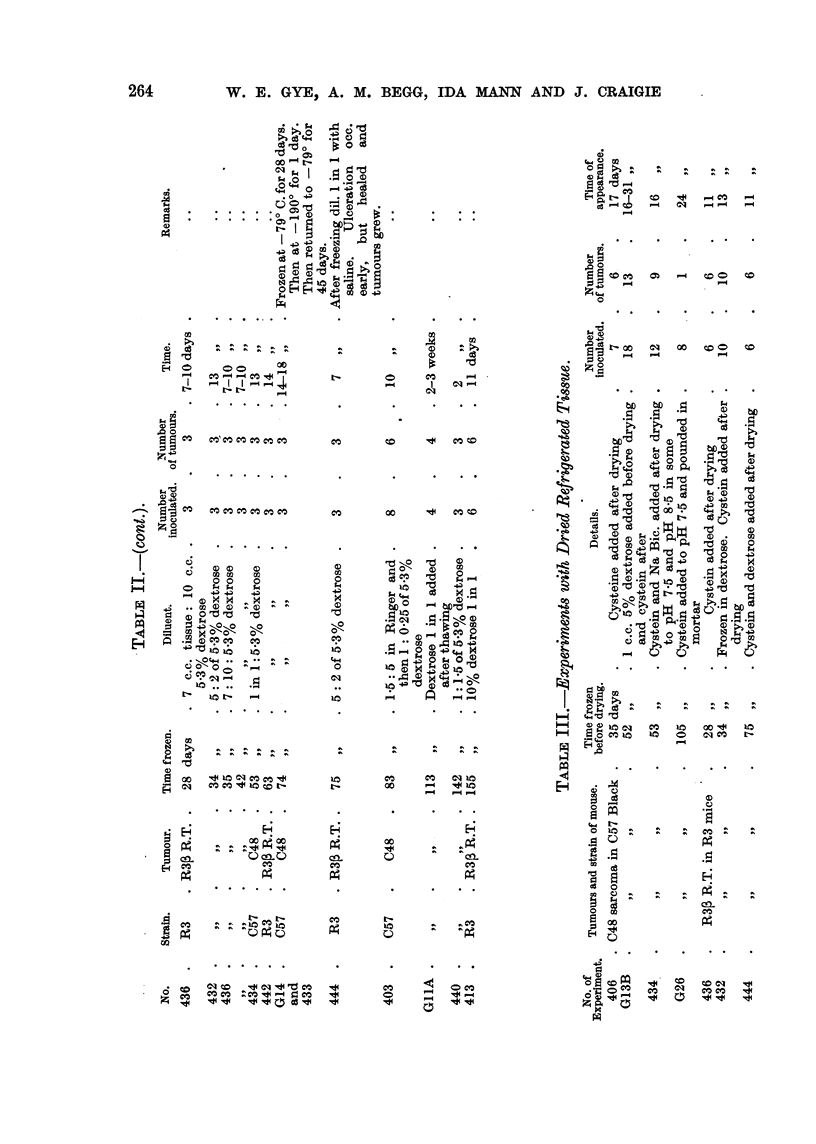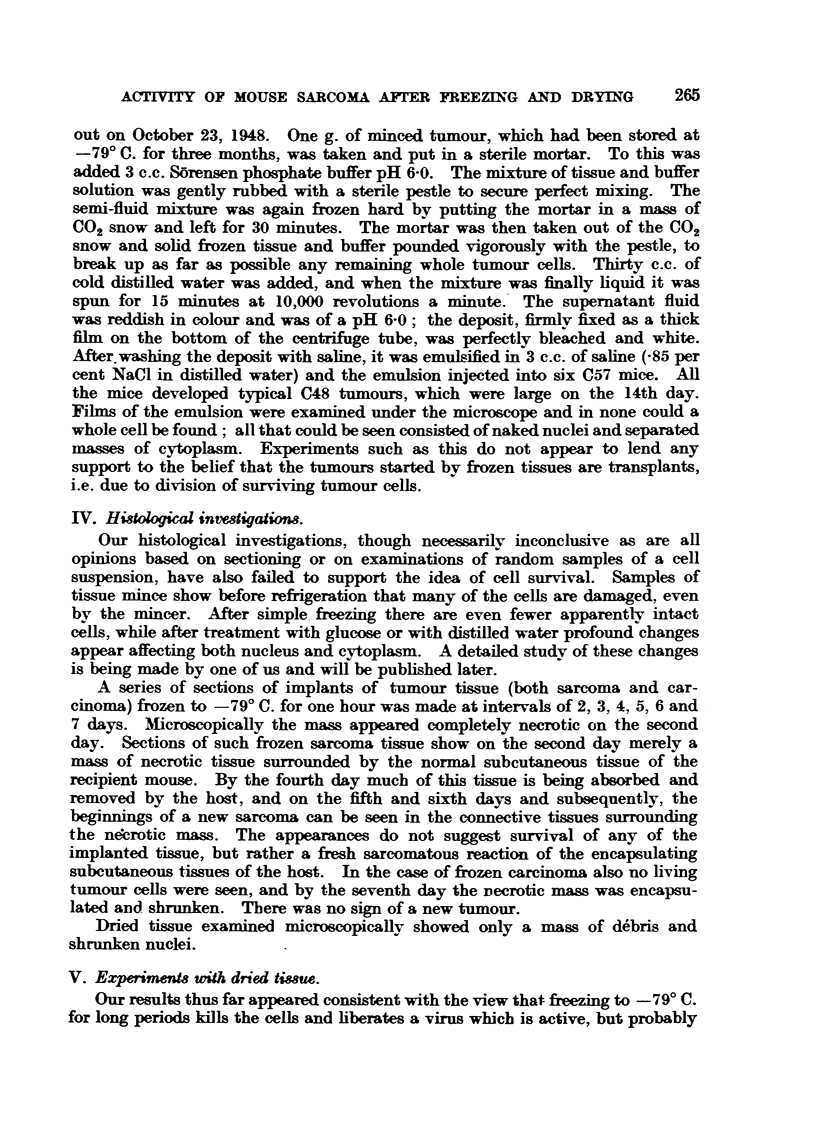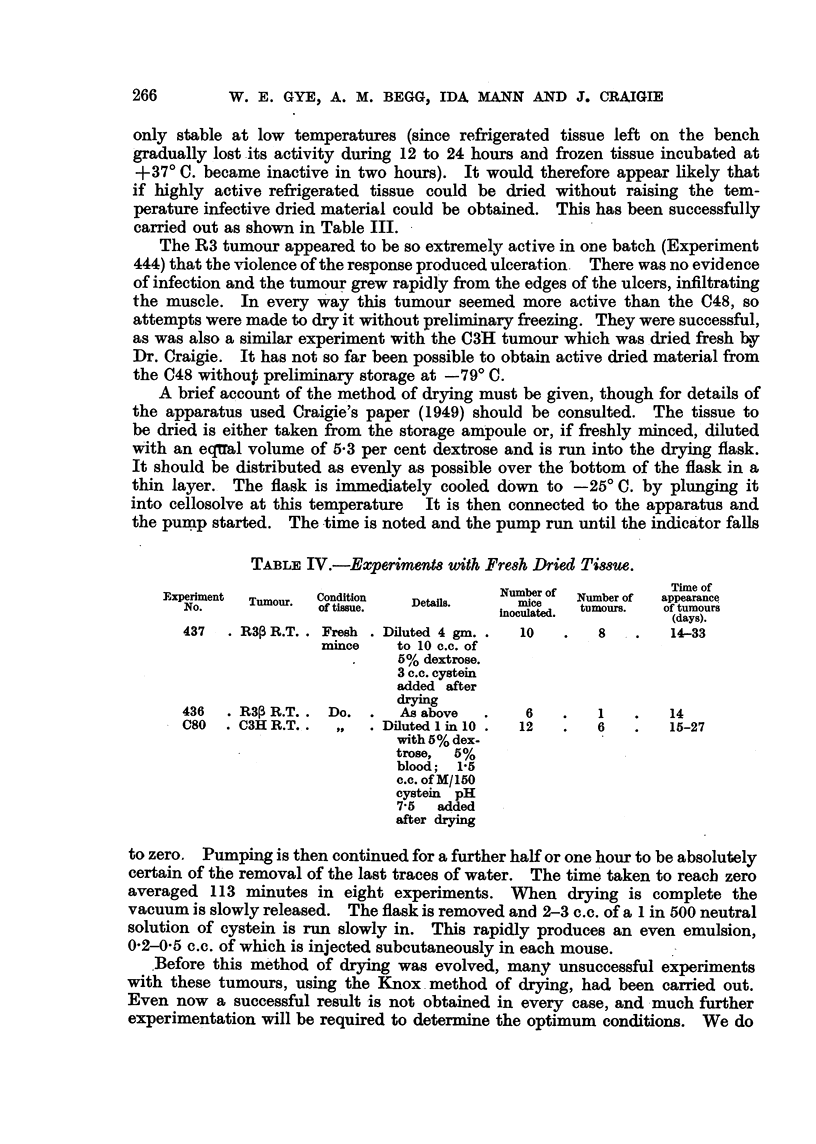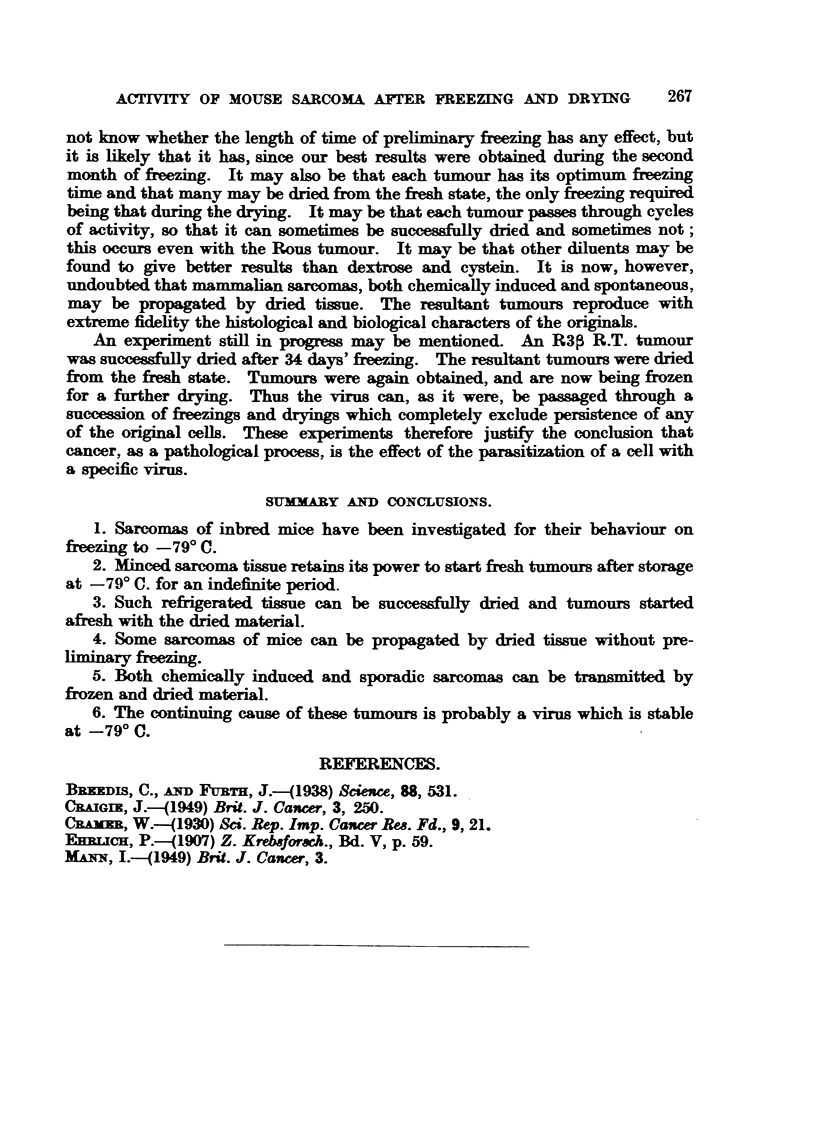# The Survival of Activity of Mouse Sarcoma Tissue after Freezing and Drying

**DOI:** 10.1038/bjc.1949.29

**Published:** 1949-06

**Authors:** W. E. Gye, A. M. Begg, Ida Mann, James Craigie


### THE SURVIVAL OF ACTIVITY OF MOUSE SARCOMA TISSUE AFTER FREEZING AND DRYING.

W. E. GYE, A. M. BEGG, IDA MANN AND JAMES CRAIGIE.

*From the Laboratories of the Imperial Cancer Research Fund, London, N.W.7.*

Received for publication March 9, 1949.

NUMEROUS authors, from Ehrlich (1907) to Breedis and Furth (1938), have reported that tumour tissue, both sarcomatous and carcinomatous, can be kept for long periods, certainly more than a year, at low temperatures from —10° C. to —79° C., without losing the power to start tumours afresh in the appropriate animals. From this and other papers in the present series it will be seen that we have amply confirmed these findings. Extremely active material capable of starting fresh tumours in less than two weeks has been obtained from both chemically induced and spontaneous sarcomas frozen at —79° C. for twelve months, and from sporadic carcinomas frozen at —79° C. for over three months. This phenomenon of survival of activity of cancer cells under conditions which are known to kill normal mammalian embryo cells (Mann, 1949) appears worthy of investigation. since it involves the question, first raised by Ehrlich in 1907, in connection with a possible bacterial source of cancer, of whether extreme cold can be used to destroy mammalian cells, and simultaneously to preserve any possible intra- or extra-cellular agent capable of starting a tumour *de novo* in cells of another host. If the continued activity of such refrigerated tissue is due to survival of cells of the original animal, then the tumour produced in the new host is merely a transplant. If, however, it can be shown that the cells in the refrigerated tissue are dead, then the new tumour must be produced by an agent (which would then be the " continuing cause " of cancer) which is a separate entity from the cells in which it lives, and which is capable of surviving under certain conditions when they are dead. Such transmission would be " cell-free," and would place any tumour so produced in the class of virus tumours, among the known virus chicken tumours, the Shope papilloma of rabbits, Bittner's mammary carcinoma and Lucké's adenocarcinoma of the frog.

The proof that prolonged refrigeration kills mammalian tumour cells is difficult, and we are aware that in the past it has always been held, except by Cramer (1930), that the survival of only a few cells in the tumour mass accounts for the tumour in the new host.

We have investigated the problem from a variety of angles and have concluded, as will appear, that the cells are dead.

In the first place it must be realized that the sarcomas of mice which we have used are three well known to laboratory workers, one a methylcholanthrene sarcoma (C.48 in the inbred strain C57 Black), and the other two arising as stromal transformations in transplants of sporadic mammary cancers investigated by R. J. Ludford (the R3β R.T. and the C3H R.T.). These three tumours have hitherto resisted all attempts at cell-free transmission, so that our results, as will be seen, point, not to the accidental discovery of three new mammalian virus tumours, but to the demonstration of a common virus origin for two whole classes (chemically induced and sporadic) of mouse tumours hitherto considered unrelated to the known virus sarcomas of birds, or to the Shope papilloma or the Bittner mammary cancer.

In the second place we have subjected the tumour tissue to various processes besides refrigeration which are known to kill mouse embryo cells, such as fine mincing and dispersion, dilution with glucose, with cystein, buffering to pH6, storage in glycerol and treatment with distilled water. None of these procedures affect the activity of the refrigerated tissue, which remains as great as or greater than that of the fresh tissue. Thirdly, we have made histological examinations of frozen tissue implants at intervals and have examined the material itself, finding, however, neither any evidence for survival of cells, nor conclusive proof of their total destruction, since one cannot be certain that every cell has been examined.

Finally, being well aware that to all these methods of approach might be opposed the objection of the remote possibility of the exceptional survival of a few cells, we have proceeded to dry the refrigerated tissue completely to dust *in vacuo* at a temperature of $-25°$ C. This is universally allowed to be lethal to both normal and tumour cells, yet we have obtained highly active dry material from all the three tumours studied. It therefore appears impossible to avoid the conclusion that from both the chemically induced and the sporadic sarcomas a cell-free agent can be recovered, which on injection into appropriate mice is capable of starting the tumour afresh. From its resistance to extreme cold, to glycerol and to drying the agent seems most likely to be a virus, and since a virus origin is undoubted for many avian sarcomas, this is less surprising than would be a non-virus origin for mammalian sarcoma.

Our experiments will now be described in detail.

I. *The tumours used.*

(1) A sarcoma (C48) of the inbred strain C57 Black. This tumour was obtained by the injection of an oily solution of methylcholanthrene in 1941 ; it has been propagated by grafting during the past seven years. During this period attempts had been made again and again to propagate the tumour with cell-free filtrates and with dry tissue, and all had previously failed.

(2) A sarcoma of the strain R3. This arose in the stroma of a spontaneous mammary carcinoma which was transplanted by R. J. Ludford. The sarcoma which formed from the stromal connective tissue was separated from the mammary cancer by Ludford by early transplantations by which the slower growing carcinoma was lost.

(3) A similar sarcoma derived from the stroma of a carcinoma of the strain C3H. This also was obtained as a pure sarcoma by Dr. Ludford.

All three tumours are soft, rapidly growing sarcomas. The R3 sarcoma is the most malignant, the methylcholanthrene tumour the least.

II. *Simple refrigeration experiments.*

The technique was similar for all the experiments. The tumour was dissected and minced, either as finely as possible with scissors or in a Craigie mincer (Craigie, 1949). The mince was drawn up in a syringe with a wide bore needle and put into sterile ampoules, 1 c.c. in each. Occasionally it was diluted with a little saline. The ampoules were then sealed, labelled and frozen to $-79°$ C. by immersion in a solution of solid $CO_2$ in cellosolve. The temperature of the ampoule was sometimes dropped slowly and sometimes rapidly. No difference in results was noticed. so that no allowance need be made for thermal shock. The ampoules were then transferred to a storage cabinet kept at $-79°$ C. After an appropriate interval of hours, days or months, an ampoule was taken out, thawed to room temperature, opened, and 0·05 c.c. or less of the contents inoculated subcutaneously in the right flank of mice of the appropriate strain. When these ampoules were opened it was always noted that a physical change had taken place in their contents, which had become more homogeneous, softer and slightly sticky after freezing. Twenty-four experiments were made with inoculations at varying intervals with uniformly good results (Table I). It will be noted that the time frozen makes no difference to the results. In the last experiment on the list the material had been frozen 201 days, and was inoculated not only into its mouse of origin, but also into four other strains. Tumours appeared in all four strains.

III. *Refrigeration in various media.*

From experiments with mouse embryo tissue (Mann, 1949) we know that treatment with glucose (without refrigeration) reduces the activity of normal mammalian cells, that storage in glycerol at 4° C. kills them, and that a combination of dextrose and cystein severely damages them, while freezing alone for one hour kills them all. Various experiments with tumour cells using glycerol, dextrose, cystein, and distilled water with and without freezing were therefore performed. Table II shows that these procedures, known to kill normal cells, have no effect on the activity of tumour tissue, dextrose indeed appearing definitely beneficial (Craigie, 1949).

It will be noted that the results in Table I and Table II are very similar, and are consistent with the presence of a tumour agent other than a living mammalian cell. As an example of the amount of manipulation which such tumour mince will stand we can cite the protocol of an experiment with the C48 tumour, carried out on October 23, 1948. One g. of minced tumour, which had been stored at $-79°$ C. for three months, was taken and put in a sterile mortar. To this was added 3 c.c. Sörensen phosphate buffer pH 6·0. The mixture of tissue and buffer solution was gently rubbed with a sterile pestle to secure perfect mixing. The semi-fluid mixture was again frozen hard by putting the mortar in a mass of $CO_2$ snow and left for 30 minutes. The mortar was then taken out of the $CO_2$ snow and solid frozen tissue and buffer pounded vigorously with the pestle, to break up as far as possible any remaining whole tumour cells. Thirty c.c. of cold distilled water was added, and when the mixture was finally liquid it was spun for 15 minutes at 10,000 revolutions a minute. The supernatant fluid was reddish in colour and was of a pH 6·0; the deposit, firmly fixed as a thick film on the bottom of the centrifuge tube, was perfectly bleached and white. After washing the deposit with saline, it was emulsified in 3 c.c. of saline (·85 per cent NaCl in distilled water) and the emulsion injected into six C57 mice. All the mice developed typical C48 tumours, which were large on the 14th day. Films of the emulsion were examined under the microscope and in none could a whole cell be found; all that could be seen consisted of naked nuclei and separated masses of cytoplasm. Experiments such as this do not appear to lend any support to the belief that the tumours started by frozen tissues are transplants, i.e. due to division of surviving tumour cells.

IV. *Histological investigations.*

Our histological investigations, though necessarily inconclusive as are all opinions based on sectioning or on examinations of random samples of a cell suspension, have also failed to support the idea of cell survival. Samples of tissue mince show before refrigeration that many of the cells are damaged, even by the mincer. After simple freezing there are even fewer apparently intact cells, while after treatment with glucose or with distilled water profound changes appear affecting both nucleus and cytoplasm. A detailed study of these changes is being made by one of us and will be published later.

A series of sections of implants of tumour tissue (both sarcoma and carcinoma) frozen to $-79°$ C. for one hour was made at intervals of 2, 3, 4, 5, 6 and 7 days. Microscopically the mass appeared completely necrotic on the second day. Sections of such frozen sarcoma tissue show on the second day merely a mass of necrotic tissue surrounded by the normal subcutaneous tissue of the recipient mouse. By the fourth day much of this tissue is being absorbed and removed by the host, and on the fifth and sixth days and subsequently, the beginnings of a new sarcoma can be seen in the connective tissues surrounding the necrotic mass. The appearances do not suggest survival of any of the implanted tissue, but rather a fresh sarcomatous reaction of the encapsulating subcutaneous tissues of the host. In the case of frozen carcinoma also no living tumour cells were seen, and by the seventh day the necrotic mass was encapsulated and shrunken. There was no sign of a new tumour.

Dried tissue examined microscopically showed only a mass of débris and shrunken nuclei.

V. *Experiments with dried tissue.*

Our results thus far appeared consistent with the view that freezing to $-79°$ C. for long periods kills the cells and liberates a virus which is active, but probably only stable at low temperatures (since refrigerated tissue left on the bench gradually lost its activity during 12 to 24 hours and frozen tissue incubated at +37° C. became inactive in two hours). It would therefore appear likely that if highly active refrigerated tissue could be dried without raising the temperature infective dried material could be obtained. This has been successfully carried out as shown in Table III.

The R3 tumour appeared to be so extremely active in one batch (Experiment 444) that the violence of the response produced ulceration There was no evidence of infection and the tumour grew rapidly from the edges of the ulcers, infiltrating the muscle. In every way this tumour seemed more active than the C48, so attempts were made to dry it without preliminary freezing. They were successful, as was also a similar experiment with the C3H tumour which was dried fresh by Dr. Craigie. It has not so far been possible to obtain active dried material from the C48 without preliminary storage at —79° C.

A brief account of the method of drying must be given, though for details of the apparatus used Craigie's paper (1949) should be consulted. The tissue to be dried is either taken from the storage ampoule or, if freshly minced, diluted with an equal volume of 5·3 per cent dextrose and is run into the drying flask. It should be distributed as evenly as possible over the bottom of the flask in a thin layer. The flask is immediately cooled down to —25° C. by plunging it into cellosolve at this temperature It is then connected to the apparatus and the pump started. The time is noted and the pump run until the indicator falls

TABLE IV.—*Experiments with Fresh Dried Tissue.*

| Experiment No. | Tumour. | Condition of tissue. | Details. | Number of mice inoculated. | Number of tumours. | Time of appearance of tumours (days). |
|---|---|---|---|---|---|---|
| 437 | R3β R.T. | Fresh mince | Diluted 4 gm. to 10 c.c. of 5% dextrose. 3 c.c. cystein added after drying | 10 | 8 | 14–33 |
| 436 | R3β R.T. | Do. | As above | 6 | 1 | 14 |
| C80 | C3H R.T. | „ | Diluted 1 in 10 with 5% dextrose, 5% blood; 1·5 c.c. of M/150 cystein pH 7·5 added after drying | 12 | 6 | 15–27 |

to zero. Pumping is then continued for a further half or one hour to be absolutely certain of the removal of the last traces of water. The time taken to reach zero averaged 113 minutes in eight experiments. When drying is complete the vacuum is slowly released. The flask is removed and 2–3 c.c. of a 1 in 500 neutral solution of cystein is run slowly in. This rapidly produces an even emulsion, 0·2–0·5 c.c. of which is injected subcutaneously in each mouse.

Before this method of drying was evolved, many unsuccessful experiments with these tumours, using the Knox method of drying, had been carried out. Even now a successful result is not obtained in every case, and much further experimentation will be required to determine the optimum conditions. We do not know whether the length of time of preliminary freezing has any effect, but it is likely that it has, since our best results were obtained during the second month of freezing. It may also be that each tumour has its optimum freezing time and that many may be dried from the fresh state, the only freezing required being that during the drying. It may be that each tumour passes through cycles of activity, so that it can sometimes be successfully dried and sometimes not; this occurs even with the Rous tumour. It may be that other diluents may be found to give better results than dextrose and cystein. It is now, however, undoubted that mammalian sarcomas, both chemically induced and spontaneous, may be propagated by dried tissue. The resultant tumours reproduce with extreme fidelity the histological and biological characters of the originals.

An experiment still in progress may be mentioned. An R3β R.T. tumour was successfully dried after 34 days' freezing. The resultant tumours were dried from the fresh state. Tumours were again obtained, and are now being frozen for a further drying. Thus the virus can, as it were, be passaged through a succession of freezings and dryings which completely exclude persistence of any of the original cells. These experiments therefore justify the conclusion that cancer, as a pathological process, is the effect of the parasitization of a cell with a specific virus.

### SUMMARY AND CONCLUSIONS.

1. Sarcomas of inbred mice have been investigated for their behaviour on freezing to −79° C.
2. Minced sarcoma tissue retains its power to start fresh tumours after storage at −79° C. for an indefinite period.
3. Such refrigerated tissue can be successfully dried and tumours started afresh with the dried material.
4. Some sarcomas of mice can be propagated by dried tissue without preliminary freezing.
5. Both chemically induced and sporadic sarcomas can be transmitted by frozen and dried material.
6. The continuing cause of these tumours is probably a virus which is stable at −79° C.

### REFERENCES.

Breedis, C., and Furth, J.—(1938) *Science*, **88**, 531.

Craigie, J.—(1949) *Brit. J. Cancer*, **3**, 250.

Cramer, W.—(1930) *Sci. Rep. Imp. Cancer Res. Fd.*, **9**, 21.

Ehrlich, P.—(1907) *Z. Krebsforsch.*, Bd. V, p. 59.

Mann, I.—(1949) *Brit. J. Cancer*, **3**.